# The Prognosis and Predictive Value of Estrogen Negative/Progesterone Positive (ER−/PR+) Phenotype: Experience of 1159 Primary Breast Cancer from a Single Institute

**DOI:** 10.1155/2022/9238804

**Published:** 2022-05-17

**Authors:** S. Gamrani, S. Boukansa, Z. Benbrahim, N. Mellas, F. Fdili Alaoui, M. A. Melhouf, C. Bouchikhi, A. Banani, M. Boubbou, T. Bouhafa, H. El Fatemi

**Affiliations:** ^1^Laboratory of Biomedical and Translational Research, Hassan II University Hospital, University Sidi Mohammed Ben Abdillah, Fez, Morocco; ^2^Department of Pathology, Hassan II University Hospital, Fez, Morocco; ^3^Department of Oncology, University Hospital Center Hassan II, Fez, Morocco; ^4^Department of Obstetrics and Gynecology, University Hospital Center Hassan II, Fez, Morocco; ^5^Department of Radiology, University Hospital Center Hassan II, Fez, Morocco; ^6^Department of Radiation Oncology, University Hospital Center Hassan II, Fez, Morocco

## Abstract

Breast cancer is a serious worldwide public health problem and is currently the most common cancer overall. Its endocrine therapy is related to the expression of the steroid hormones, estrogen receptor (ER), and progesterone receptor (PR). Breast cancers can be presented under multiple profiles of steroid hormones: ER(−)/PR(+), ER(+)/PR(−), double-positive/negative ER, and PR. 2–8% of all breast cancers express only PR (ER−/PR+) which is an abnormal phenotype, with less known about their behaviors and outcomes. Our study was performed on a large and well-characterized database of primary breast cancer from 2012 to 2019, up to 1159 cases. These cases were divided according to ER and PR expression, as we put all of our focus on ER-negative/PR-positive group, more specifically ER−/PR+/HER2+ and ER−/PR+/HER2− gene expressions, to highlight their features and find a pattern that links HR (hormone receptors) profiles and breast cancer subtypes. Out of the informative cases, 94 patients (8%) had ER−/PR+ breast cancers, while 676 (58.4%) had ER+/PR+, 88 (7.6%) had ER+/PR−, and 164 (14.2%) had ER−/PR− tumors. The ER−/PR+ group was statistically correlated with a high risk of recurrence and death in midway between the double-negative and double-positive HR. According to HER2 status, a low DFS was observed in patients ER−/PR+/HER2−, which is closer to the DFS of TNBC cases but worse than ER+/PR any. On the other side, the ER−/PR+/HER2+ showed also a poorer DFS closer to the HER2+ subgroup in between TNBC and ER+/PR any. The clinicopathological features of the ER−/PR+/HER2− and ER−/PR+ HER2+ have distinguished the patients into two groups with a difference in some clinicopathological characteristics: both groups had closer OS estimation, which was worse than ER−/PR any and better than TNBC and HER2. The ER−/PR+/HER2− seems to increase the risk of recurrence than ER−/PR+/HER2+ when compared to ER+/PR any. On the other hand, the ER−/PR+/HER2+ seems to increase the risk of death more than ER−/PR+/HER2− in comparison with ER+/PR any. Our results support that ER−/PR+ tumors really exist and are rare and clinically and biologically distinct subtypes of breast cancer. In addition, our analysis, which was based on dividing the groups according to HER2 expression, has revealed the existence of two distinct groups; this gave the ER−/PR+ subgroup a heterogeneity characterization. Moreover, this breast cancer subtype should not be treated as a luminal tumor but rather according to the HER2 expression status.

## 1. Introduction

Breast cancer is a serious worldwide public health problem and is currently the most common cancer overall [1], causing the highest number of cancer-related deaths among women. Due to its complexity and heterogeneity, breast cancer presents veritable variation in clinical, morphological, and molecular management [2]. The molecular classification by immunohistochemical expression of estrogen receptor ER, progesterone receptor PR, human epidermal growth factor receptor 2 (HER2), and proliferation index Ki-67 established by St. Gallen surrogate for breast cancer subtypes reveals five main entities: luminal-A, luminal-B HER2-negative, luminal B HER2-positive, HER2 enriched, and TNBC (triple negative: lack of expression of ER, PR, and no overexpression of HER2) [3, 4].

Breast cancer endocrine therapy is actually related to the expression of the steroid hormones, estrogen receptor (ER), and progesterone receptor (PR). The estrogen receptor (ER) and progesterone receptor (PR) are expressed in more than 75% of breast cancers [5, 6]. They are one of the most powerful prognostic factors and predictive markers in hormonal treatment [7–9]. Therefore, breast cancers can be presented in multiple profiles of steroid hormones: ER(−)/PR(+), ER(+)/PR(−), double-positive/negative ER, and PR [3].

The treatment strategies decisions in cases of double-positive/negative steroid hormones can be taken easily [9]. Not to mention, hormone-receptor-positive breast tumors are qualified by less aggressive clinicopathological outcomes and a high prognosis in reason of the benefits from endocrine therapy [10].

Estrogen receptors (ER) status on its own is useful in predicting benefits from antiestrogenic treatment, but not from hormonal treatment. Thus, progesterone receptors are often tested in parallel with estrogen receptors, as studies have shown that PR expression is conditional on ER activity [3, 4, 11, 12]. Consequently, the luminal tumors are the most common breast cancer phenotypes, presenting more than 50% of all breast cancers [9]. Moreover, only 15–20% of all breast cancer cases have expressed one hormone receptor at a time, with a predominance of tumors expressing ER, but not PR (ER+/PR−) [13, 14].

The existence of breast cancer with ER-negative/PR-positive phenotype is still debated. The biological significance, prognosis, and predictive impact of ER−/PR+ breast cancers have been discussed; there are some hypotheses about considering this profile as a technical artifact. The HR status of breast cancer may be altered due to several factors, resulting in a false-negative ER and/or false-positive PR assay. Antibody selection for ER testing, improper tissue fixations, and different thresholds for reporting immunostaining or less sensitive immunohistochemistry, are some of these factors [15–17]. The American Society of Clinical Oncology/College of American Pathologists recommended that ER−/PR+ tumors should be tested repeatedly to avoid false negative ER results [7, 18].

In response, some authors raised an important issue: this special profile (ER−/PR+) could represent a distinct and unique entity. ER−/PR+ breast tumors have different behaviors and patient characteristics when compared to double-positive/negative ER and PR tumors. Several studies have demonstrated that ER−/PR+ tumors appear more commonly in younger and premenopausal women and are associated with more aggressive behavior than ER+/PR+ disease [7].

In this retrospective cohort, we investigated a well-characterized database of primary breast cancer cases from 2012 to 2019. Our aim was to describe the clinical features and outcomes of estrogen receptor-negative (ER−) and progesterone receptor-positive (PR+). The cases were divided according to ER and PR expression. So, we put our focus on ER-negative/PR-positive group to highlight their features and to figure any prognosis and predictive value in comparison with ER+/PR−, double-positive/negative, luminals, HER2 enriched, and triple-negative breast cancer.

## 2. Patients and Methods

### 2.1. Patients and Treatments

Our study was based on a consecutive series of 1159 cases of primary invasive breast cancer patients diagnosed at HASSAN II University Hospital Center of Fez between 2012 and 2019. Clinicopathological data were collected from the pathologic database of the laboratory of anatomic pathology of HASSAN II University Hospital of Fez. We excluded all patients with missed hormone receptor results, in situ carcinoma, and other breast nonepithelial tumors.

Specimen were obtained through biopsies in metastatic cases and through biopsies and surgical resections for nonmetastatic cases. Surgery was mainly radical mastectomy (Patey) or conservative surgery. All cases have been discussed in the multidisciplinary tumor board for deciding about (neo)-adjuvant treatment. All the decisions about radiotherapy, chemotherapy, hormone therapy, or targeted therapy conform with the European Society of Medical Oncology Guidelines [19].

### 2.2. Histopathological Analysis

The histological analysis has been performed on formalin-fixed and paraffin-embedded tissue sections, with hematoxylin-eosin-saffron staining. The histological grade assessment of tumors was established according to the Nottingham Histological Score system [20].

### 2.3. Immunohistochemistry Assessment

All of our patients had the ER, PR, and HER2 status and KI67 expression. Immunohistochemical analysis was performed on paraffin-embedded tissues from the breast primary tumors, by immunohistochemical strainers (Ventana BenchMark LT from 2009 to 2011 and Ventana BenchMark ULTRA from 2012 to 2019), using primary antibodies according to the manufacturer's guidelines. At our pathology department, positive and negative controls were routinely performed, including the processing of normal tissue or tumor sections. Receptor statuses were reported prospectively, and HR expression was defined according to ASCO/CAP guidelines (2020), low positive (1–10%), and negative (0 or < 1%). For patients with 0 stainings, HR evaluation was repeated twice and then considered to have negative expression. For the proliferation index KI67, it was the overall average and we chose a cut-off of 20% to evaluate the positivity expression of KI67 (high: KI67 > 20%, low KI67 < 20%). HER2 was assessed using immunohistochemistry or fluorescence in situ hybridization FISH. Moreover, Immunohistochemical scores of 0 or 1+ were defined as negative and scores of 3+ were defined as positive. For tumors scored 2+, they were tested using FISH and the positive result was defined based on HER2 to CEP17 ratio over 2 according to (ASCO/CAP 2007, 2013, 2018) guidelines.

### 2.4. Statistical Analysis

Statistical analysis was performed using SPSS 23 statistical software. We evaluated the association between the ER−/PR+ profile and other clinicopathological features using the chi-square test, and Fisher's exact test as appropriate. The survival curves were performed using the Kaplan–Meier method and Cox regression to evaluate prognostic markers. We considered tests as statistically significant when *p* < 0.05.

### 2.5. Follow-Up

In nonmetastatic breast cancer cases, regular follow-up visits were organized every 3–4 months in the first 2 years, every 6 months from years 3 to 5, and then annually with annual mammography and regular bone density evaluation for patients receiving AIs or ovarian suppression. In metastatic breast cancer cases, follow-up was done every 12 weeks with clinical examination and chest-abdomino-pelvic CT scan.

Overall survival (OS) duration was defined as the time between the date of diagnosis and death resulting from breast cancer or the last follow-up visit, death, being scored as an event. Disease-free interval (DFI) was calculated from the date of the first diagnosis and the date of first distant or local disease recurrence or last follow-up being scored as an event.

## 3. Results

**Figure 1:**
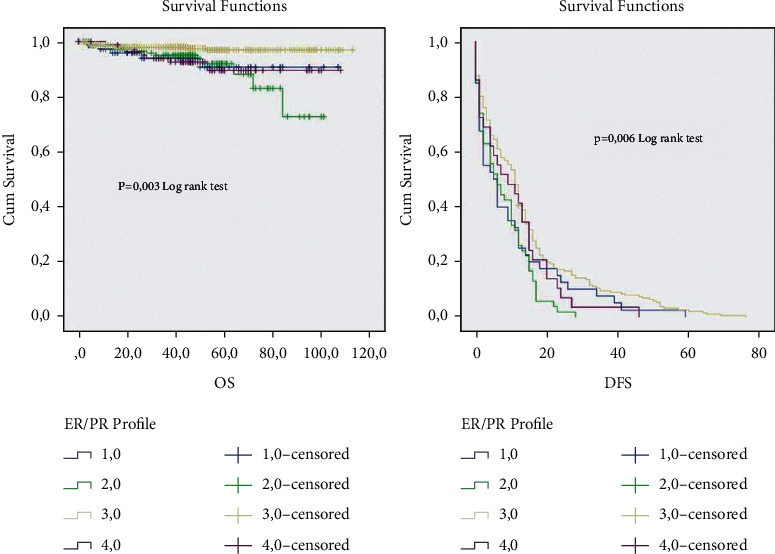
OS overall survival and DFS diseases free survival rates according to ER and PR status in all patients. 1-ER−/PR+; 2-ER−/PR−; 3-ER+/PR+; 4-ER+/PR−.

1. Clinicopathological and therapeutic characteristics of patients
2. On the total of 1159 patients treated, all of them were women, with a mean age of 49 years and with a majority (69%) of patients with an age less than 50 years. Invasive breast carcinoma of no special type was the most histological type in our study. Most patients, up to 50%, were grade II, 32% of the rest were grade III, and then came 10.4% of patients who were grade I. Regarding TNM classification, 40% of patients are classified T2, 15% T3, and 26% T4. A great number of patients had a tumor size more than 2 cm (74%). The lymph nodes metastasis was positive for the majority of tumors. Regarding the immunohistochemical analysis, the hormone receptor expression showed a positivity expression for ER and PR and a high proliferation index in a larger number of patients. (Neo)-adjuvant treatments were decided according to the ESMO guidelines. From the enrolled cases, 14% of patients received neo-adjuvant chemotherapy, 62.3% received adjuvant chemotherapy, 56.4% received hormone therapy (tamoxifen only, AI only, or tamoxifen concurrent with AI), and 53.6% were treated with radiotherapy.
3. Clinicopathological characteristics of ER−/PR+ group in relation to the other ER/PR profiles expression
4. In the current retrospective study of primary breast cancer, the median age at diagnosis was 48 (17–88) years. The clinicopathological characteristics of patients are listed in Table 1. Out of the informative cases, 94 patients (8%) had ER−/PR+ (ER < 1% and PR > 1%) breast cancers, while 676 (58.4%) had ER+/PR+ (ER > 1% and PR > 1%), 88 (7.6%) had ER+/PR− (ER > 1% and PR < 1%), and 164 (14.2%) had ER−/PR− (ER < 1% and <PR 1%) tumors. 619 (53%) patients were younger at the time of diagnosis (≤50 years), and there was a significant statistical difference between the groups (*p*=0.05); in comparison, the ER+/PR+ and ER−/PR+ were younger at the time of diagnosis (55% and 51%, respectively) than ER+/PR− and ER−/PR− (60% and 50%, respectively) (≥50 years). 542 patients (53%) had grade II, 363 (35.0%) grade III, and 108 (10.0%) grade I. A significant difference across hormone receptor profiles (*p* ≤ 0.001) was observed. Furthermore, double-positive hormone receptors and ER+/PR− groups had the highest frequency of Grade II (55.3% and 46%, respectively), while most of the patients with ER−/PR− (56%) and ER−/PR+ (50%) had Grade III. Out of all subjects, there were 376 (57.9%) patients who had positive significant association to lymph node status (*p*=0.038). The majority of patients were significantly HER2+ and had high proliferation index (*p*=0.008 and *p*=0.002, respectively), Table 2.
5. The relationship between the adjuvant therapy and the recurrence in the enrolled patients
6. Clinicopathological Characteristics of ER−/PR+ profile and the different breast cancer molecular subtypes
7. The analyzed profiles of HR expression were differed by age at the time of diagnosis. The ER−/PR+/HER2− (54%) and the ER+/PR any (56%) and HER2+ (53%) patients were younger (≤50 years) while the ER−/PR+/HER2+ (56%) and the TNBC (54%) patients were older (>50 years). However, no statistical significance was shown (*p* value = 0.2). Almost all patients showed a very statistically significant correlation to high grade III; on the contrary, a big number of ER+/PR any patients were grade II. No significant correlation was obvious to tumor size, *p*=0.1, even though all patients presented a high tumor size (tumor size >2). Indeed, the lymph nodes status was statistically correlated (*p*=0.001) and the percentage of negative lymph nodes status was higher in ER−/PR+/HER2− and TNBC. On the other hand, the positive lymph node status was more often in ER−/PR+/HER2+, ER+/PR any, and HER2+ tumors. All patients were statistically related to a high ki67 expression (*p*=0.034) (Table 3).
8. Overall survival and diseases free survival rates according to ER and PR status in all patients
9. The survival data analysis shows a median follow-up period of 108,2 months (range 0–113 months). A remarkable significant difference was perceived between the four hormone receptors curves (ER−/RP+, ER−/PR−, ER+/PR+, ER+/PR−) in terms of overall survival and diseases free survival (OS: log-rank = 13, 96, *p*=0.003; and DFS: log-rank = 12, 53, *p*=0.006, respectively). However, the ER−/PR− and ER+/PR- profiles present approximate OS and DFS rates (DFS: 10.2 (6–14); 10.7 (7–14), and OS: 99.6 (93–105); 100 (94–105), respectively), which showed outcomes midway between double negative (poor prognosis) and double positive (good prognosis) (Table 4 and Figure 1).

**Figure 2:**
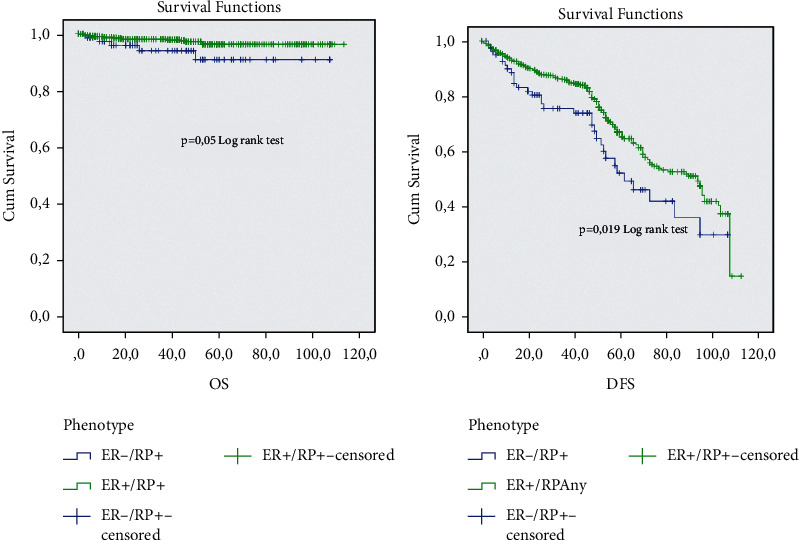
Overall survival and diseases free survival analysis of the ER−/PR+ and ER+/PR any subgroup.

**Figure 3:**
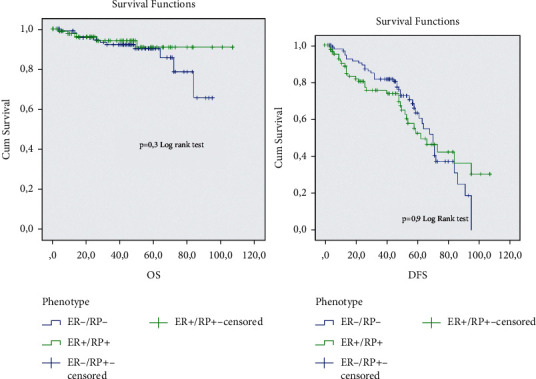
Overall survival and diseases free survival analysis of the ER−/PR+ and double negative HR subgroup.

**Figure 4:**
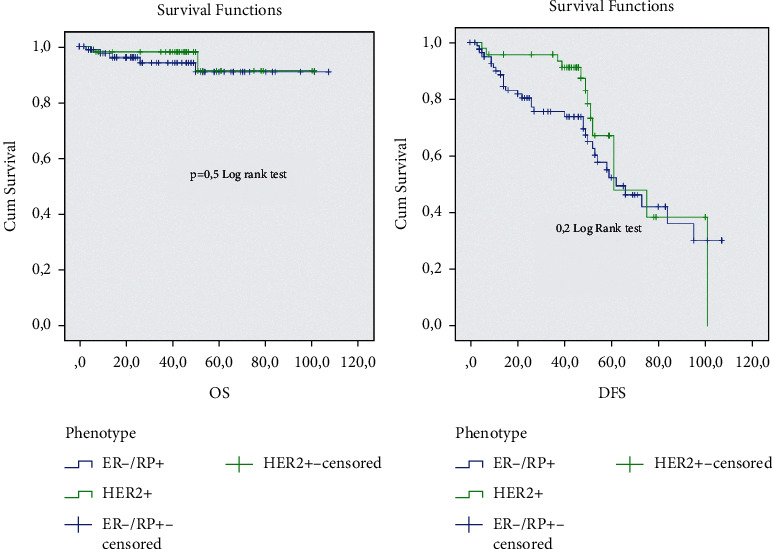
Overall survival and diseases free survival analysis of the ER−/PR+ and HER2+ subgroup.

The analysis of the prognostic impact of the ER−/PR+ subgroup in comparison with different ER/PR groups shows that this subgroup has a significantly short overall survival and short disease survival in comparison with the ER+/PR any (*p*=0.05). The ER−/PR+ subgroup shows a good prognosis in comparison with the TNBC and HER2+ subgroup, but no significant *p*was observed (Figures 2–4).

**Figure 5:**
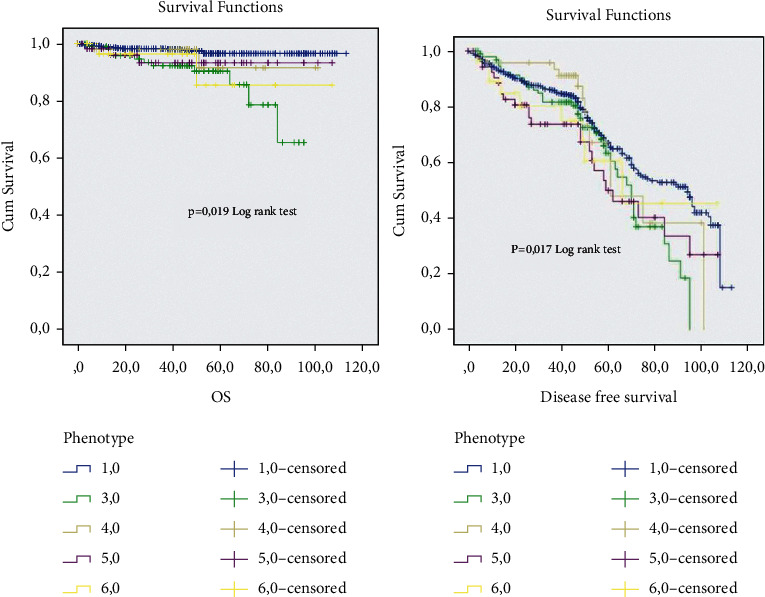
Relation among 5-ER−/PR+/HER2−, 6-ER−/PR+/HER2+, 1-ER+/PR any, 3-TNBC, and 4-HER2+ and disease-free interval in the whole series; (b) relation among 5-ER−/PR+/HER2−, 6-ER−/PR+/HER2+, 1-ER+/PR any, 3-TNBC, and 4-HER2+ and overall survival in the whole series.

The analysis of the whole breast cancer cases showed an important association between the breast cancer subtypes and outcomes (OS: log-rank = 5.5, *p*=0.017, and DFS: log-rank = 5.7, *p*=0.019, respectively). However, a low DFS was observed in patients ER−/PR+/HER2−, which is closer to the DFS of TNBC cases (Estimation 64 vs. 65, respectively) but worse than ER+/PR any. Furthermore, the ER−/PR+/HER2+ showed a poor DFS closer to HER2+ subgroup (70 vs. 71) and between TNBC as poorer prognosis and ER+/PR any as best prognosis (77) DFS. Additionally, the OS estimations of the ER−-/PR+/HER2− and ER−/PR+ HER2+ were closer (100 vs. 97) to classify them in the midway of the ER+/PR any and TNBC, HER2+. The ER−/PR+/HER2− seems to increase the risk of recurrence even more than ER−/PR+/HER2+ compared to ER+/PR any. For death, the ER−/PR+/HER2+ seems to increase the risk than ER−/PR+/HER2− in comparison with ER+/PR any (Table 5 and Figure 5).

## 4. Discussion

Steroid hormones play a critical role in the assessing and the management of breast cancer; they are an important prognostic and predictive biomarker [8]. The existence of ER−/PR+ phenotype remains uncertain and this may make it difficult to determine an appropriate treatment.

In our research, 8% of patients have presented the ER−/PR+ phenotype; this is consistent with the previously published cohort using ER and PR IHC [3]. These women were younger than ER+/PR any and almost have the same age of ER−/PR− subgroups at the time of diagnosis and exhibited less favorable prognostic factors compared to women with ER+ disease, including higher grade, more frequent nodal metastases, HER2 overexpression/amplification, and high KI67 expression. The association between the ER−/PR+ profile and younger age has been supported by laboratory data and previous studies, showing that PR expression is more common in premenopausal women diagnosed with primary breast cancers [7]. Moreover, as to breast cancer subtypes, similar results were reported by Rakha et al., in 2007, who found that patients with estrogen receptor (ER)-negative/progesterone reception (PR)-positive and ER-positive/PR-negative tumors are distinct breast cancer phenotypes; they have more advanced clinicopathological features when compared with the double-positive and favorable feature when compared with double-negative breast cancers. Focusing on the ER−/PR+, Leen et al., Yunhai Li et al., and Melissa et al. [7, 16, 21], reported a similar phenomenon in those patients with the single hormone receptor-positive subtype who had more aggressive clinicopathological features. The fact that ER−/PR+/HER2-patients were younger at the time of diagnosis like ER+/PR any and HER2+, with high SBR grade III like TNBC HER2+, high tumor size and negative lymph node status like TNBC, and that ER−/PR+/HER2+ patients were older at the time of diagnosis like TNBC, with high SBR grade differing from ER+/PR any, high tumor size but with positive lymph node status, reveals that ER−/PR+/HER2− and ER−/PR+ HER+ are distinct ER−/PR+ breast cancer phenotypes with distinct clinicopathological characteristics.

We have found an important association between the breast cancer subtypes and outcomes (OS: log-rank = 5.5, *p*=0.017, and DFS: log-rank = 5.7, *p*=0.019, respectively). However; the low DFS rate of ER−/PR+/HER2− and ER−/PR+/HER2+ closer respectively to TNBC and HER2+ characterized them as poorer prognosis than ER+/PR any. Additionally, the OS estimations of the ER−/PR+/HER2− and ER−/PR+ HER2+ were closer (100 vs. 97) to classify them in the midway of the ER+/PR any and TNBC, HER2+. The ER−/PR+/HER2− seems to most increase the risk of recurrence than ER−/PR+/HER2− compared to ER+/PR any. For death, while ER−/PR+/HER2+ seems to increase the risk of death more than ER−/PR+/HER2− in comparison with ER+/PR any, a study by Bae et al. reported that single positive hormone receptor profiles are associated with poorer disease-free survival and OS than double-positive hormone receptor tumors, but they found that single positive hormone receptor tumors are associated with poor survival similar to that of the double negative hormone receptor subtype in Her2− negative BC although there was no difference in survival among the 4 subtypes in patients with Her2− positive disease [22]. In contrast, double-positive hormone receptor subtypes showed better DFS and OS. These results are consistent with other studies [21–24]. According to several studies, patients with the double-positive HR subtype had a better prognosis than patients with the double-negative HR. Nonetheless, the prognosis of single hormone receptor-positive BC remains unknown [9, 13, 21, 23]. Furthermore, Anderson et al. [23] classified ER-positive/PR-positive, ER-positive/PR-negative, ER-negative/PR-positive, and ER-negative/PR-negative subtypes from good to worse according to BCSS; yet, this study has some limitations in weakening the robustness of the funding. Rakha et al. [9] revealed no statistically significant survival difference between the single positive hormone receptors or between double negative and single positive hormone profiles.

In summary, we have explored the biological characteristics and outcomes of the ER−/PR+/HER2+ and ER−/PR+/HER2− subtype; this result supports that ER−/PR+ exists and is a rare tumor. The results also indicate that ER-negative/PR-positive tumors are distinct subtypes of breast cancer. Moreover, after analyzing the groups according to HER2 expression, we reveal two distinct groups which give the ER−/PR+ subgroup a heterogeneity characterization; this breast cancer subtype should not be treated as a luminal tumor but rather according to the HER2 expression status. This funding needs further studies and clinical trials to explore behavioral characteristics of single hormone receptors in order to optimize the treatment management for patients with single ER−/PR+ BC.

## Figures and Tables

**Table 1:** Relationship between the adjuvant therapy and the recurrence.

Adjuvant therapy	Recurrence 95% confidence interval	*p* value
Hormonal therapy		0.004
Yes	78.31 (74.44–82.17)	
No	72.772 (67.62–77.91)	
Chemotherapy		0.000
Yes	71.287 (67.81–74.76)	
No	85.663 (79.84–91.48)	
Radiation		0.000
Yes	77.76 (73.82–81.70)	
No	71.98 (67.01–76.95)	
Neo-adjuvant chemotherapy		0.040
Yes	68.45 (61.38–75.53)	
No	77.231 (74.00–80.45)	

**Table 2:** The clinicopathological characteristics of the four ER/PR profiles of included patients.

	ER+/RP+ (58.4%)	ER+/RP− (7.6%)	ER−/RP− (14.2%)	ER−/RP+ (8.1%)	*p* value
Characteristics					
Age groups at diagnosis					*p*=0.05
≤50 years	369 (55.4%)	36 (41%)	77 (49.7%)	48 (51.1%)	
>50 years	297 (44.6%)	52 (60%)	78 (50.3%)	46 (48.9%)	

Grade					*p* ≤ 0.001
1	78 (11.7%)	7 (8%)	2 (1.3%)	10 (10.6%)	
2	368 (55.3%)	41 (46.6%)	55 (33.5%)	32 (34%)	
3	165 (24.8%)	31 (35.2%)	87 (56.1%)	47 (50%)	
NA	(8.2%)	10.2%	9%	5.4%	

Tumor size					*p*=0.2
≤2 cm	185 (36.3%)	22 (33.8%)	30 (24.2%)	18 (28.6%)	
>2 cm	305 (59.8%)	42 (64.6%)	89 (71.8%)	43 (68.3%)	
NA	3.9%	1.6%	4%	3%	

Lymph node status					*p*=0.038
Positive	301 (45%)	38 (43%)	57 (40.2%)	29 (31.2%)	
Negative	142 (21.24%)	23 (26.1%)	53 (36.8%)	23 (24.5%)	
NA	66.2%	31%	23%	44%	

PtT	149 (22.1%)	17 (19.3%)	29 (17.7%)	15 (16%)	*p*=0.8
T2	243 (36.1%)	32 (36.4%)	76 (46.3%)	27 (28.7%)	
T3	33 (5%)	8 (9.1%)	9 (5.5%)	7 (7.4%)	
T4	27 (4%)	5 (5.7%)	5 (3%)	7 (7.4%)	
NA	32%	29.5%	27.5%	40.5%	

HER2 status					*p*=0.008
Negative	467 (20.7%)	48 (38.6%)	97 (31.6%)	54 (34%)	
Positive	138 (70.6%)	33 (53.4%)	49 (62%)	33 (59.6%)	
NA	8.7%	8%	6.4%	6.4%	

KI67					*p*=0.002
≤20%	155 (28.6%)	16 (29.1%)	19 (17.9%)	11 (16%)	
>20%	387 (72%)	39 (70.9%)	87 (82.1%)	65 (84%)	

NA: not available.

**Table 3:** Clinicopathological characteristics of ER−/PR+ and the breast cancer subtypes.

	ER−/PR+/HER2−	ER−/PR+/HER2+	ER+/PR any	Her2+	TNBC	*p* value
Characteristics						
Age groups at diagnosis						*p*=0.2
≤50 years	33 (54.1%)	15 (44.1%)	342 (56.2%)	26 (53.1%)	45 (45.5%)	
>50 years	28 (45.9%)	19 (55.9%)	267 (43.8%)	23 (46.9%)	54 (54.5%)	

Grade						*p* ≤ 0.0001
1	8 (13.1%)	2 (5.9%)	77 (12.5%)	0 (0%)	1 (1%)	
2	21 (34.4%)	11 (32.4%)	344 (56.2%)	23 (49%)	26 (26.3%)	
3	28 (45.9%)	141 (58.8%)	158 (25.9%)	24 (51.0%)	63 (63.6%)	
NA	4 (6.6%)	1 (2.9%)	30 (4.9%)	4 (4.1%)	9 (9.1%)	

Tumor size						*p*=0.1
≤2 cm	13 (31.7%)	9 (37.5%)	167 (34.8%)	9 (23.1%)	18 (21.7%)	
>2 cm	26 (63.4%)	15 (62.5%)	291 (60%)	28 (71.0%)	62 (74.7%)	
NA	2 (4.9%)	0	22 (4.6%)	2 (5.1%)	3 (3.6%)	

Lymph node status						*p*=0.001
Negative	18 (29.5%)	7 (22%)	141 (23%)	17 (34.7%)	41 (41.4%)	
Positive	16 (26.2%)	14 (41.1%)	279 (45.7%)	18 (37%)	30 (30.4%)	
NA	27 (44.2%)	13 (38.2%)	190 (31%)	14(28%)	28 (28.3%)	

pT						*p*=0.1
T1	9 (14.8%)	7 (20.7%)	128 (21%)	9 (18.4%)	13 (13%)	
T2	17 (27.9%)	12 (35.3%)	236 (38%)	21 (43%)	50 (50.5%)	
T3	6 (9.8%)	1 (2.9%)	33 (5.4%)	3 (6.1%)	6 (6.1%)	
T4	4 (6.6%)	3 (8.8%)	26(%)	1 (2%)	3 (3%)	
TX	25 (41%)	11 (32.4%)	183 (30%)	15 (30.6%)	25 (25.3%)	

KI67						*p*=0.034
KI67 < 20%	9 (18%)	3 (10.7%)	166 (27.7%)	7 (20.6%)	10 (15.2%)	
KI67 > 20%	41 (82.3%)	25 (89.3%)	433 (72.3%)	27 (79.4%)	56 (84.8%)	

NA: not available.

**Table 4:** League table of comparison.

	Mean (DFS)	*p* value	Mean (OS)	*p* value
1-ER−/PR+	10.2 (6–14)	=0.006	99.6 (93–105)	=0.003
2-ER−/PR−	7.7 (5–10)		91 (84–96)	
3-ER+/PR+	14.2 (12–6)		108 (108–111)	
4-ER+/PR−	10.7 (7–14)		100 (94–105)	

**Table 5:** League table of comparison.

	Mean (DFS)		*p* value	Mean (OS)		*p* value
	Estimation	95% CI		Estimation	95% CI	
5-ER−/PR+/HER2−	64	52.775.3	0.017	100	94–107	0.019
6-ER−/PR+/HER2+	70	52–88.5		97	84–110	
1-ER+/PR any	77	74–81		110	108–111	
3-TNBC	65	58.5–71.2		83	77.5–89.8	
4-HER2	71.9	59–84		95.7	88.5–103	

## Data Availability

Data are available on request.
